# Inequalities in the economic and social cost of food allergy

**DOI:** 10.1186/2045-7022-1-S1-S73

**Published:** 2011-08-12

**Authors:** Lynne Regent

**Affiliations:** 1 The Anaphylaxis Campaign, Farnborough, Hampshire, UK

## 

The economic and social impact of food allergy affects many sectors of society.There are associated costs to individuals,their carers and their households,potentially over a lifetime. In the health sector resourses are required for diagnosis,support and education.The entire food chain is affected through extra costs,food regulations and moral obligations.Also the employment sector is affected in terms of lost productivity. There is currently a lack of information and clear methodology for assessing the economic and social cost of food allergy to the individual and to society.Hardest to quantify are the costs associated with quality of life factors such as health,financial security,standard of living, family and friends and general well-being.The Europrevall Project attempts to address these issues and acknowledges that further work in this area is required. It is possible to ilustrate the the social and economic impact of food allergy from the perspective of the individual and their families and carers through an examination of the issues regularly brought to the attention of patient support groups.These may not be easily quantifiable but are nonetheless very real.These impacts can be alleviated by ensuring that individuals and families receive the services they need and through improved understanding,awareness and training. Undoubtedly more research is needed in this area.Patient groups need to continue to support people coping with these challenges and to ensure that the requirement for education and training across the public and private sectors is recognised.

